# Step-by-Step to Achieve our Goals

**DOI:** 10.5935/1678-9741.20160067

**Published:** 2016

**Authors:** Domingo M. Braile

**Affiliations:** 1 Editor-in-Chief - BJCVS

Dear Colleagues,

We have always been working hard to keep the Brazilian Journal of Cardiovascular Surgery (BJCVS) active and up-to-date, despite the uncertainties that pervade the political, economic, social and scientific activities in our country.

Those who did not imagine the evil influence that would be exerted by the instability that has taken place in the public or private Universities, were wrong. The negative impact on teaching and research, caused by a lack of resources, was enormous.

Much more serious was the impact on hospitals, medical clinics, primary healthcare centers, Intensive Care Units (ICUs), as well as on health equipment, dependent on federal, state, municipal or private funding, due to the widespread undercapitalization of governments and the population, seriously damaging the patient care, and the data generation, essential to the research.

The question that won't go away is: What does the BJCVS have to do with all this background?

Unfortunately, we have a lot to do with it, because the manuscripts that come to us are basically from "Stricto or Lato Sensu" postgraduate programs, related to patient care, and to a lesser extent regarding the basic research.

In the Editorial Issue 31-1 of the BJCVS, I pointed out that we were facing a great challenge in 2016, due to the many changes and adjustments implemented during this period. I was concerned about the flow of manuscripts and their quality, could be enough to celebrate the journal's 30th anniversary with dignity, getting a better Impact Factor (IF).

In this issue we have the intelligent answer to my apprehension.

## BJCVS 30 YEARS

In the section BJCVS 30 YEARS, the brilliant Professor Paulo Evora gives us a lesson on the Impact Factor (IF) and its ills.

I ask you to carefully, read the article: Brazilian Journal of Cardiovascular Surgery 30 years: Does, the Impact Factor, matter? (Page 266).

By reading this article, we can appreciate not only the author's opinion, as well as other subject-matter experts cited by him, showing how flawed the valuation processes are, performed by Thompson Reuters and Scimago exclusively in order to guide the booksellers. However, it was adopted in Brazil to generate the Qualis, which is maintained by CAPES, a methodological excrescence that harms the publications of countries like Brazil, and also the specialties, which are unlikely to have as many citations as a generalist journal who has a huge field of authors and readers.

At the beginning of the article, Professor Evora expresses my hope, in the paragraph that should always be repeated:

"Brazil is much greater than the crises it faces. It is essential to show what we are capable of in order to stand amidst other nations". (Domingo M. Braile, 2016).

I hope this optimism becomes a reality.

In the same section, there are also two excellent articles; one of them, by the Professor Walter J. Gomes: Thirtieth anniversary of the Brazilian Journal of Cardiovascular Surgery. And devising the next decades (page 265)

The author presents us with a futuristic vision of hope, only seen in patriotic leaders.

Another article that is also worth reading is: 30 Years of BJCVS (page 267)

Written by Professor João de Deus e Brito, who besides doing a very well prepared historical review, shows his deep knowledge of the BJCVS, whose he has always been a dedicated reviewer.

I thank the collaboration of these professors on my own behalf and in the name of the whole Editorial Board and Technical Clerks, who continue the saga and the tradition of our journal.

## ASSOCIATE EDITORS

As I mentioned at the beginning of this Editorial, we have been working with a focus on modifications that were suggested to improve the performance of our journal.

For now, were indicated seven Associate Editors, in the areas of coronary diseases, valve diseases, congenital diseases, aorta diseases, advances in cardiovascular surgery and basic research.

The choice criteria were absolutely based on meritocracy, dependent on the performance of the Associate Editors and Reviewers over the years.

The task was made easier by the ongoing assessment performed by the journal's staff for many years.

The selection of the Area Editors, and the areas themselves, is not finished yet, and should be perfected throughout the time.

## SUBMISSION SYSTEM

As almost everyone knows, we are migrating to the: submission, review, approval, or rejection of the manuscript to ScholarOne platform, developed by Thomson Reuters and adopted by SciELO, with FAPESP's economic support.

This platform is internationally considered ideal for the globalized journals.

The deadline for the definite migration to the new platform is scheduled to August 15th.

I hope the implantation time can be fulfilled, since all the work is being developed with great professionalism.

## EVENTS

Among the good news about the events, I emphasize some very important factors:

The participation of the SBCCV with a special session during the 71st Congress of the SBC in Fortaleza, on September 23, 2016.

In the 44th Congress of SBCCV the main theme will be: High Technology: (acquiring knowledge to employ it better), to be held in Rio de Janeiro, on April 20-22, 2017 with special participation of international speakers such as Prof. Eric J. Velazquez (Durham, USA); Prof. Hartzell V. Schaff (Minnesota, USA); Prof. John Puskas (New York, USA); Prof. Michael M. Madani (San Diego, USA) and Prof. Manuel Antunes (Coimbra, Portugal).

## ARTICLES

As you will see, we have good national and international articles in this issue, with notable articles from China - Mild Hypothermia May Offer Some Improvement to Patients with MODS after CPB Surgery (page 246); Fungal Endocarditis (page 252); One from Romania - Primary Aortoduodenal Fistula: First you Should Suspect it (page 261).

With great satisfaction, we received the first article from Portugal, Endovascular Abdominal Aneurysm Repair in Women: What are the Differences Between the Genders? (Page 232).

I wish you all a very nice and instructive read.

I thank, as always, the essential support of the Board of Directors, Associate Editors, Technical Staff and especially the Authors and Readers who keep the BJCVS alive.

Warmest regards,

Domingo M. Braile Editor-in-Chief - BJCVS

